# Factors Associated With Postoperative Arrhythmias in Patients Undergoing Isolated Off-Pump Coronary Artery Bypass Grafting: A Single-Center Prospective Observational Study

**DOI:** 10.7759/cureus.106776

**Published:** 2026-04-10

**Authors:** Gurpreet Panesar, Nirja P Patel, Kunal A Soni, Kartik B Dhami, Manish Tiwari, Vishal V Bhende

**Affiliations:** 1 Cardiac Anaesthesiology, Bhanubhai and Madhuben Patel Cardiac Centre, Shree Krishna Hospital, Bhaikaka University, Karamsad, IND; 2 Cardiac Surgery, Bhanubhai and Madhuben Patel Cardiac Centre, Shree Krishna Hospital, Bhaikaka University, Karamsad, IND; 3 Pediatric Cardiac Surgery, Sri Sathya Sai Sanjeevani Centre for Child Heart Care and Training in Pediatric Cardiac Skills, Navi Mumbai, IND; 4 Pediatric Cardiac Surgery, Bhanubhai and Madhuben Patel Cardiac Centre, Shree Krishna Hospital, Bhaikaka University, Karamsad, IND

**Keywords:** atrial fibrillation, dexmedetomidine, hemodynamics, off pump coronary artery bypass grafting, postoperative arrhythmias

## Abstract

Background and aim

Postoperative arrhythmia is very common after isolated coronary artery bypass grafting (CABG). Indeed, it is the most frequently encountered arrhythmia and an important contributor to postoperative morbidity and mortality. This study aimed to evaluate the incidence of postoperative arrhythmias and to identify factors associated with their occurrence in patients undergoing isolated off-pump CABG.

Methods

This prospective observational study was conducted on 100 patients who underwent isolated CABG at Bhanubhai and Madhuben Patel Cardiac Centre from January 2020 to September 2021. Demographic and hemodynamic data were recorded intraoperatively and postoperatively. The primary outcome was postoperative arrhythmia, and atrial fibrillation was analyzed as a subtype. Bivariate analysis followed by multivariate logistic regression was performed to identify factors associated with postoperative arrhythmia. Adjusted odds ratios (OR) with 95% confidence intervals (CI) were reported.

Results

The incidence of postoperative arrhythmia was 23%, of which atrial fibrillation occurred in 17% and ventricular premature complexes in 6% of patients. Univariate analysis revealed that age, diabetes mellitus, and intraoperative bradycardia were associated with the occurrence of arrhythmia. On multivariate logistic regression, diabetes mellitus (OR 3.31; 95% CI 0.97-11.27), intraoperative bradycardia (OR 4.76; 95% CI 1.06-21.27), and intraoperative tachycardia (OR 8.89; 95% CI 1.09-72.27) were associated with postoperative arrhythmia.

Conclusions

Diabetes mellitus and intraoperative hemodynamic variations were associated with postoperative arrhythmias in patients undergoing off-pump CABG. These findings represent associative relationships and should be interpreted cautiously given the observational design and limited sample size.

## Introduction

Despite advances in anesthesia techniques and improved cardiac surgical skills, perioperative arrhythmia remains common after cardiac surgery [1]. Atrial tachyarrhythmia is more common in the postoperative period than ventricular arrhythmias and bradyarrhythmias. The incidence of postoperative atrial fibrillation (AF) after isolated coronary artery bypass grafting (CABG), valve surgery, and combined valve and CABG procedures is 15%-40%, 37%-50%, and 60%, respectively [2]. Postoperative AF can lead to hemodynamic instability, thromboembolic phenomena, mortality and morbidity, and prolonged stays in the intensive care unit (ICU) and the hospital, thereby increasing the overall cost of treatment [3]. Accordingly, the prevention of postoperative AF has attracted considerable attention recently [4].

Off-pump CABG differs from on-pump CABG in terms of inflammatory response, myocardial ischemia-reperfusion injury, and autonomic imbalance, all of which may influence the occurrence of postoperative arrhythmias. However, most existing prediction models for postoperative atrial fibrillation are derived from mixed or on-pump CABG populations. Therefore, studying arrhythmias specifically in off-pump CABG patients may help identify perioperative factors relevant to this subgroup. The aim of the present study was to identify the factors associated with AF after isolated CABG.

## Materials and methods

This study was conducted at Bhanubhai and Madhuben Patel Cardiac Centre from January 2020 to September 2021 (data collection period) after approval from the Institutional Ethics Committee (IEC-2) of Bhaikaka University, Anand, vide letter No. IEC/HMPCMCE/115/Faculty/22/220/2025 dated 01/07/2025. Ethics committee approval dated 01/07/2025 was obtained for retrospective analysis of the prospective observational study and publication of the collected data.

Inclusion criteria

All patients between the ages of 30 and 80 years undergoing isolated CABG were included in the study after providing written informed consent. Consecutive eligible patients undergoing isolated off-pump CABG during the study period were included.

Exclusion criteria

Patients with severe left ventricle (LV) dysfunction (ejection fraction <30%), end-stage renal disease, hepatic dysfunction, a history of arrhythmia, or those posted for emergency or redo surgery were excluded from the study.

Anesthesia and pain management

Anesthesia induction was done with an injection of 2 mcg/kg fentanyl and 0.05 mg/kg midazolam, followed by 0.2-0.3 mg/kg etomidate. With loss of eyelid reflex, an injection of 1.0-1.5 mg/kg rocuronium was administered, and orotracheal intubation was performed. Anesthesia was maintained with top-up doses of fentanyl and midazolam, with rocuronium and sevoflurane as the inhalation agent. Intraoperative monitoring included a 12-lead electrocardiogram (ECG), pulse oximetry, invasive blood pressure monitoring, central venous pressure, and temperature monitoring. An infusion of dexmedetomidine was started after induction of anesthesia at a rate of 0.2-0.5 mcg/kg/hour, titrated as per hemodynamics, and continued in the ICU until 24 hours after initiation of the infusion. Dexmedetomidine activates central α receptors, leading to sedation, reduced anxiety, analgesia, and anti-arrhythmic activity [5].

Accordingly, perioperative infusion of dexmedetomidine is our institutional protocol for all patients undergoing off-pump coronary artery bypass surgery (OPCAB).

Definitions and methodology

Consistent with the American Heart Association (AHA) guidelines, AF was diagnosed based on ECG characteristics lasting ≥30 seconds, showing absence of P waves and an irregular R-R interval when atrioventricular conduction was present, as well as irregular atrial activity known as "fibrillatory waves" [6].

Outcome

The primary outcome of the study was the occurrence of postoperative arrhythmia during the intraoperative period and within the first 72 hours postoperatively. Atrial fibrillation was analyzed as a subtype of postoperative arrhythmia. Postoperative AF was diagnosed using an ECG showing the absence of P waves and irregular R-R intervals lasting ≥30 seconds, consistent with AHA guidelines. The secondary outcome was to assess the association of risk factors with the occurrence of postoperative AF. 

Intraoperative hypotension was defined as systolic blood pressure (SBP) <100 mm Hg. Hypotension was treated with volume therapy, reduction of dexmedetomidine infusion by 50%, and, if persistent, initiation of noradrenaline.

SBP >150 mmHg was recorded as hypertension and treated by adjusting the depth of anesthesia. When hypertension persisted, nitroglycerine was started. A reduction in heart rate to <50 beats per minute was recorded as bradycardia and treated by reducing dexmedetomidine infusion. A heart rate >100 beats per minute was recorded as tachycardia and managed by increasing the depth of anesthesia and titration of dexmedetomidine infusion.

All patients were monitored with continuous ECG in the ICU for at least 72 hours postoperatively. A daily 12-lead ECG was performed, and an additional ECG was obtained if arrhythmia was clinically suspected. Hemodynamics were recorded every 30 minutes after induction of anesthesia in the operating theater, hourly for up to six hours after transfer to the ICU, and twice hourly for up to 24 hours after initiation of dexmedetomidine infusion. The pain score was recorded for all patients undergoing OPCAB using the visual analog scale (VAS) every six hours after extubation by the duty doctor in the ICU. Patients were classified as hypotensive, hypertensive, bradycardic, or tachycardic if more than 70% of recorded readings fell into the respective category. This classification was used to reflect sustained hemodynamic exposure rather than isolated readings.

An injection of 1 g paracetamol was administered every six hours as first-line analgesia. For VAS scores >3, a second-line injection of 50 mg tramadol was administered three times daily. An infusion of 1-2 mcg/kg/hr intravenous fentanyl was started only when VAS scores exceeded 3 after tramadol administration.

Intraoperative and postoperative serum electrolytes were recorded, and hypokalemia was defined as serum potassium <3.5 mEq/L.

Statistical analysis

Data were entered into Microsoft Excel (Microsoft Corporation, Redmond, Washington) and analyzed using STATA 14.2 (StataCorp LLC, College Station, Texas). Continuous variables were expressed as mean ± standard deviation and categorical variables as frequency and percentage. Bivariate analysis was performed using Student’s t-test for continuous variables and Fisher’s exact test for categorical variables. Variables with p <0.10 in bivariate analysis were included in the multivariate logistic regression model. Multivariate logistic regression was performed to identify factors associated with postoperative arrhythmia, and adjusted odds ratios with 95% confidence intervals were reported. A p-value <0.05 was considered statistically significant. Given the limited number of outcome events, the number of variables included in the multivariate model was restricted to avoid model overfitting.

## Results

The incidence of postoperative arrhythmia was 23%. The baseline demographic variables are shown in Table 1.

**Table 1:** Baseline characteristics of the study population and comparison between patients with and without postoperative arrhythmias Continuous variables are expressed as mean ± standard deviation and categorical variables as number (percentage). P-values were calculated using Student’s t-test for continuous variables and Fisher’s exact test for categorical variables. BMI, body mass index; VAS, visual analog score.

Variable	Overall (n = 100)	Arrhythmia (n = 23)	No Arrhythmia (n = 77)	p-value
Age (years), mean ± SD	60.23 ± 9.65	63.78 ± 9.20	59.16 ± 9.59	0.04
Male, n (%)	88 (88%)	22 (95.7%)	66 (85.7%)	0.28
Female, n (%)	12 (12%)	1 (4.3%)	11 (14.3%)	
BMI (kg/m²), mean ± SD	—	—	—	0.44
Diabetes mellitus, n (%)	38 (38%)	13 (56.5%)	25 (32.5%)	0.05
Hypertension, n (%)	57 (57%)	11 (47.8%)	46 (59.7%)	0.37
Bradycardia, n (%)	22 (22%)	9 (39.1%)	13 (16.9%)	0.01
Tachycardia, n (%)	11 (11%)	4 (17.4%)	7 (9.1%)	0.10
Hypotension, n (%)	11 (11%)	4 (17.4%)	7 (9.1%)	0.37
Hypokalemia, n (%)	28 (28%)	6 (26.1%)	22 (28.6%)	1.00
VAS > 3, n (%)	37 (37%)	12 (52.2%)	25 (32.5%)	0.13

**Figure 1:**
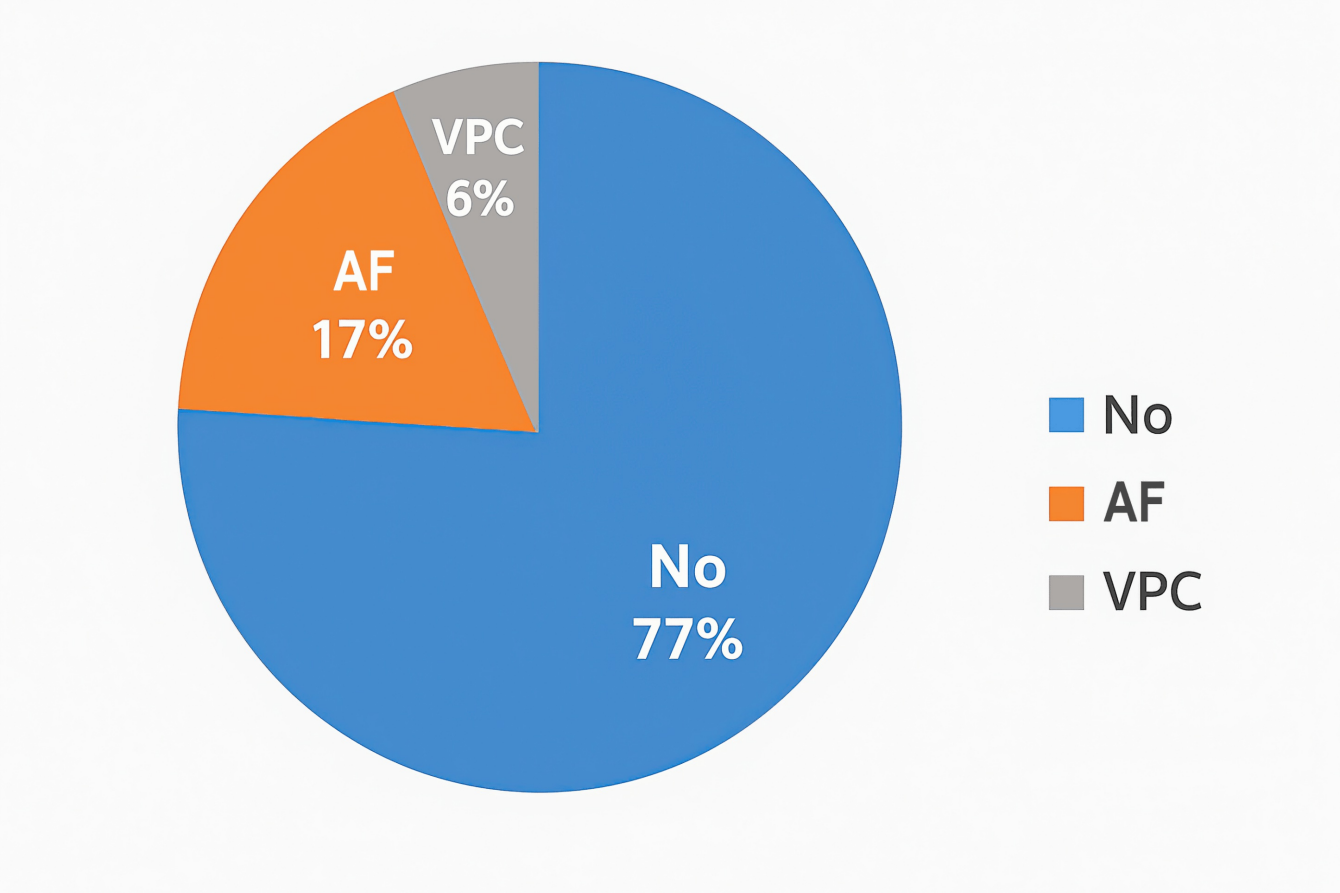
Occurrence and type of arrhythmias AF, atrial fibrillation; VPC, ventricular premature complexes.

Twenty-three of 100 patients developed postoperative arrhythmia, of which atrial fibrillation occurred in 17 patients and ventricular premature complexes occurred in 6 patients, as shown in Figure 1. 

**Figure 2:**
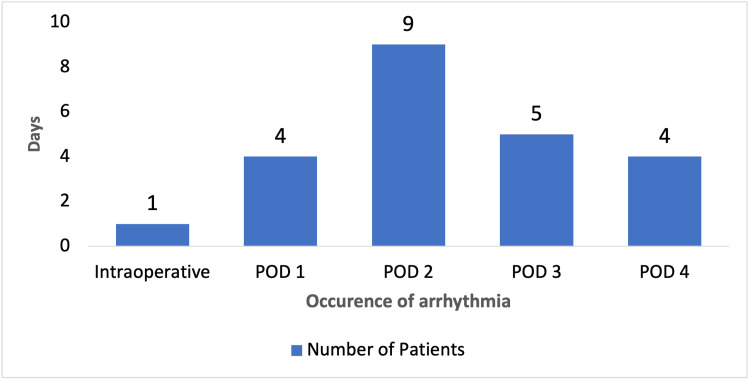
Time of occurrence of arrhythmias POD, postoperative day.

None of the patients had premature atrial complexes, ventricular tachycardia, ventricular fibrillation, or bradyarrhythmia. Of the 23 patients who developed arrhythmia, 4 had intraoperative arrhythmia (17.4%), and 4 had arrhythmia on postoperative day one (POD-1). Two of those who had arrhythmia on POD-1 had AF, and 2 had ventricular premature contractions. Nine patients had arrhythmia on the second POD and 5 on the third POD, as shown in Figure 2.

The study patients who developed postoperative arrhythmia (POA) were assigned to one of two groups. Twenty-three patients belonged to the group that developed POA (POA+), and 77 patients belonged to the group that did not develop POA (POA−).

The mean age of patients in the POA+ group was 63.78 ± 9.2 years, and that of the POA− group was 59.16 ± 9.59 years. A significant association was found between age and the occurrence of postoperative arrhythmia (p<0.05) (Table 2).

**Table 2:** Multivariate analysis of factors associated with postoperative arrhythmias The reference range/reference category for each variable is provided in the Materials and Methods section. OR, odds ratio; CI, confidence interval; K+, potassium; VAS, visual analog score [7].

Associated Variables	Category	OR	95% Cl for OR	Adjusted p-value	Unadjusted p-value
Age		1.04	0.96, 1.11	0.27	0.04
Gender	Male	2.26	0.22, 22.83	0.48	0.28
Obesity		0.94	0.81, 1.09	0.44	0.37
Hypertension	Present	1.65	0.50, 5.46	0.4	0.47
	Absent (reference category)				
Diabetes	Present	3.31	0.97, 11.27	0.05	0.05
	Absent (reference category)				
Heart rate	Bradycardia	4.76	1.06, 21.27	0.04	0.01
	Normal (reference category)				
	Tachycardia	8.89	1.09, 72.27	0.04	0.1
	Normal (reference category)				
Blood pressure	Hypertension	0.39	0.10, 1.50	0.17	0.59
	Normal (reference category)				
	Hypotension	0.51	0.06, 4.22	0.54	0.46
	Normal (reference category)				
K^+^ level	Hypokalemia	1.06	0.09, 11.51	0.95	>0.05
	Normal (reference range)				
VAS score	>3	3.03	0.93, 9.84	0.06	0.13
	<3 (reference range)				

In the POA+ group, 88% of the patients were male and 12% were female. No association was found between gender and the occurrence of arrhythmia (p<0.05).

Using the World Health Organization classification of obesity, 52 patients belonged to the category of normal body mass index (BMI) (18.5-24.19 kg/m²). The proportions of overweight and obese patients in the POA+ group were 4% and 3%, respectively, and those in the POA− group were 27% and 9%, respectively. No significant association was found between BMI and the occurrence of arrhythmia. Diabetes mellitus was found in 56.5% of the patients in the POA+ group, whereas only 32.5% of the patients in the POA− group were diabetic. A statistically significant association was found between diabetes mellitus and the occurrence of arrhythmia. No significant association was found between hypertension or other comorbidities and the occurrence of perioperative arrhythmia.

For each patient, 28 hemodynamic readings were recorded, and patients were classified as hypotensive or hypertensive when more than 70% of readings fell into the respective category, consistent with the definition provided above. Sixty-seven patients maintained a heart rate in the optimal range. Of the 22 patients who had episodes of bradycardia, 9 developed postoperative arrhythmia. However, only 4 of the 11 patients in the tachycardia group developed arrhythmia. A statistically significant association was found between bradycardia and the occurrence of arrhythmia, but not between tachycardia and arrhythmia.

Of the 57 patients who were categorized as hypertensive, 11 belonged to the POA+ group. However, 4 of the 11 patients in the hypotensive group had arrhythmia. No statistically significant association was found between either hypotension or hypertension and the occurrence of arrhythmia, or between postoperative arrhythmia and serum electrolyte levels. For the 12 patients with postoperative arrhythmia who had VAS scores exceeding 3, an infusion of 1 mcg/kg/hr fentanyl was started as third-line analgesia. However, no association was found between arrhythmia and VAS scores.

A univariate analysis was performed to evaluate the relationship between independent variables and the occurrence of arrhythmia. The univariate model revealed that age, diabetes mellitus, and intraoperative bradycardia were significantly associated with the occurrence of arrhythmia. On multivariate logistic regression analysis, diabetes mellitus (OR 3.31; 95% CI 0.97-11.27), intraoperative bradycardia (OR 4.76; 95% CI 1.06-21.27), and intraoperative tachycardia (OR 8.89; 95% CI 1.09-72.27) were associated with postoperative arrhythmia. Age was not independently associated with postoperative arrhythmia after adjustment.

## Discussion

Postoperative arrhythmia after coronary artery bypass surgery is a frequent sequela, and AF is the most common form, with an incidence of 30% [8]. AF usually occurs on the second or third postoperative day and leads to hemodynamic instability, thus increasing the risk of embolic stroke, escalating costs, and increasing mortality [9]. Preoperative factors such as increased age, reduced ventricular function, left atrial enlargement, diabetes mellitus, and obesity predispose patients to postoperative AF [10]. Additionally, intraoperative hemodynamic disturbances causing myocardial insults, electrolyte imbalance, and increased sympathetic tone contribute to the pathogenesis of postoperative arrhythmia [11].

The role of dexmedetomidine in preventing the new onset of postoperative AF is multifactorial. First, dexmedetomidine contributes to stabilizing myocardial properties by reducing ischemia-reperfusion injury [12]. Second, it has been shown to exhibit anti-inflammatory effects in many clinical trials and, thus, limits the occurrence of arrhythmia due to altered atrial physiology secondary to inflammation [13]. Lastly, it reduces catecholamine-induced triggering of AF and accentuates vagomimetic activity, thereby leading to increased repolarization and a longer refractory period [14,15] and, in turn, preventing AF. We continued dexmedetomidine infusion as well as beta blockers perioperatively to obtain an accurate picture of postoperative arrhythmia. All patients received dexmedetomidine infusion at a dose of 0.2-0.5 mcg/kg/hour after induction in all myocardial revascularization surgeries, as part of the institutional standard of care.

We found the incidence of postoperative arrhythmia to be 23%, with AF occurring in 17% of cases and VPC in 6% of cases. These findings are comparable to those of a randomized clinical trial by Ling et al., who reported an incidence of AF of 18% in the group receiving dexmedetomidine [16]. However, these researchers reported a much lower incidence of ventricular arrhythmia (0.9%). The type of arrhythmia and its incidence in various studies are shown in Tables 3, 4 [5,12,17-23].

**Table 3:** Type of arrhythmia and its incidence in various studies AF, atrial fibrillation; VPC, ventricular premature complexes; APC, atrial premature complexes; VT, ventricular tachycardia; VF, ventricular fibrillation.

Type of Arrhythmia	Present Study	Soltani et al. [17] (n = 38)	Azimaraghi et al. [18] (n = 38)	Narisawa et al. [5] (n = 16)	Ren et al. [12] (n = 81)	Turan et al. [19] (n = 765)	Karaman et al. [20] (n = 31)	Herr et al. [21] (n = 148)	Shehabi et al. [22] (n = 152)	Liu et al. [23] (n = 44)
AF	17 (17 %)	3 (7.8 %)	1 (2 %)	1 (6.3 %)	1 (1.23%)	16.3 %	2 (6.4 %)	12 (8 %)	31 (20.4 %)	6 (13.6 %)
VPC	6 (6 %)	8 (21.0%)	-	-	10 (12.34 %)	-	-	-	-	-
APC	0	6 (15.7 %)	-	-	-	-	-	-	-	-
VT	0	1 (2.6 %)	0	-	1 (1.23 %)	-	-	0	2 (1.3%)	-
VF	0	2 (5.2 %)	-	-	-	-	-	-	1 (0.7 %)	-
Brady arrhythmias	0	-	-	-	-	-	-	-	-	-

**Table 4:** Comparison of age, gender and incidence of arrhythmia in various studies AF, atrial fibrillation.

Study	Number of Patients	Age Range (Years)	Mean Age (Years)	Male	Female	Incidence of AF
Present study	100	30-80	60.23 ± 9.66	88 (88%)	12 (12 %)	17 (17 %)
Soltani et al. [17] (n = 38)	76	30-85	60.3 ± 7.1	13 (34.21%)	25 (65.78 %)	3 (7.8 %)
Azimaraghi et al. [18] (n = 38)	100	40-70	55.32 ± 8.99	38 (76%)	12 (24 %)	1 (2 %)
Narisawa et al. [5] (n = 16)	45	60-85	71.3 ± 6.2	12 (75%)	4 (25 %)	1 (6.3 %)
Ren et al. [12] (n = 81)	162	<75	60 ± 4	31%	69 %	1 (1.23 %)
Turan et al. [19] (n = 765)	765 (duration: 5 years)	>18	58 ± 15	70%	30 %	16.3 %
Karaman et al. [20] (n = 31)	64	40-75	62.5 ± 6.8	26 (83.8%)	5 (16.2 %)	2 (6.4 %)
Herr et al. [21] (n = 148)	295	-	61.9 ± 9.5	137 (93%)	11 (7 %)	12 (8 %)
Shehabi et al. [22] (n = 152)	306	≥60	71.5 (66-76)	114 (75%)	38 (25 %)	31 (20.4 %)
Liu et al. [23] (n = 44)	90	>18	53 (46-63)	21 (47.72%)	23 (52 %)	6 (13.6 %)

Shehabi et al. reported the incidence of postoperative AF to be 20.4% in the dexmedetomidine group, which is higher than the incidence that we observed. This discrepancy may be attributable to a larger sample size (152 compared with 100 in our study) and a higher mean age (71.5 years compared with 60.2 years in our study) [22].

Contrary to our findings, Liu et al. [23] and Azimaraghi et al. [18] found the incidence of postoperative AF to be 13.6% and 2%, respectively, significantly lower than the incidence observed in our study. The discrepancy between our study and these previous studies may be attributable to the smaller sample size in our study (44 compared with 100 and 50 compared with 100, respectively), younger patients (mean age of 53 years compared with 60.26 years, and 55.3 years compared with 60.23 years), and higher doses of dexmedetomidine (0.2-1.5 mcg/kg/hour compared with 0.2-0.5 mcg/kg/hour). In our study, the univariate logistic model showed that age, diabetes mellitus, and intraoperative bradycardia were significantly associated with the occurrence of arrhythmia.

In contemporary advanced health services and preventive medicine, the mean age of patients who present with coronary artery disease is higher than in the past. However, contemporary patients tend to be sicker, with a high incidence of comorbidities, and are therefore at increased risk of morbidity and mortality [24]. Thus, in a review of 915 adult patients in sinus rhythm who underwent valve surgery, the odds ratio for developing postoperative AF was 1.51 per decade [25]. Obesity, diabetes mellitus, and AF are interlinked. Left atrial enlargement, ventricular remodeling, elevated plasma volume, autonomic tone, ventricular diastolic dysfunction, and increased neurohormonal activation can cause electrical dysfunction and, thus, predispose to postoperative atrial fibrillation [26].

We found, using a univariate logistic model, that diabetes mellitus was an independent risk factor for the development of postoperative AF. Perrier et al. also found diabetes mellitus to be an independent risk factor for the development of postoperative AF [27]. However, contrary to our findings, these researchers also found a BMI of 35 kg/m² or higher to be a predictor of AF. This discrepancy between our study and theirs may be attributable to the relatively small number of patients with a high BMI in our study.

We measured hemodynamics for each patient from induction through 24 hours in the ICU. Patients were assigned to either the bradycardia group or the tachycardia group depending on which group accounted for more than 70% of the readings. Persistent bradycardia may be a manifestation of an underlying conduction system abnormality or may be iatrogenic, being associated with medications used to treat AF or heart failure [28]. Symptomatic bradycardia can progress to AF and may require placement of a permanent pacemaker. The incidence of bradycardia was higher in our study (22%) compared with the dexmedetomidine arms of studies such as Karaman et al. [20] and Herr et al. [21]. In the current study, the univariate logistic model revealed that bradycardia had a statistically significant association with postoperative AF.

Atrial tachycardia can occur as a result of hypoxia, ischemic heart disease, pulmonary disease, use of caffeine, cocaine, chocolate, or alcohol, metabolic disturbances, digoxin toxicity, or heightened sympathetic tone. All types of paroxysmal supraventricular tachycardia can trigger AF, a condition commonly known as “tachycardia-induced tachycardia.” After the onset of atrial tachycardia, sudden dilation of the atria can occur, altering membrane potential and leading to AF [29]. Rotter et al. reported that coronary sinus tachycardia led to AF [30].

In our study, multivariate logistic regression showed the association of bradycardia, tachycardia, and diabetes mellitus with the occurrence of postoperative arrhythmia. Age was not found to be associated with AF in the multivariate model because of the close range of ages of patients in both groups. The strength of our study is the prospective observational design, which ensured the accuracy of the collected data. Since all surgeries were isolated OPCAB performed by a single surgeon, the duration and tissue handling were similar across cases.

The present study identified diabetes mellitus, intraoperative bradycardia, and intraoperative tachycardia as factors associated with postoperative arrhythmia. However, due to the observational design, these associations should not be interpreted as causal relationships. The wide confidence intervals observed in the regression analysis likely reflect the limited number of outcome events and possible model instability.

Limitations 

This study has several limitations. First, the sample size was small, with a limited number of outcome events, which may have resulted in model overfitting and wide confidence intervals. Second, this was a single-center study, which may limit generalizability. Third, all patients received dexmedetomidine, which may act as a confounding factor. Fourth, echocardiographic parameters and inflammatory markers were not included. Fifth, the study evaluated only early postoperative arrhythmias and did not assess long-term outcomes.

## Conclusions

In this prospective observational study of patients undergoing isolated off-pump CABG, diabetes mellitus and intraoperative hemodynamic variations were associated with postoperative arrhythmias. These findings represent associations rather than causal or predictive relationships and should be interpreted cautiously. Larger multicenter studies are required to validate these findings.

## References

[1] Villareal RP, Hariharan R, Liu BC (2004). Postoperative atrial fibrillation and mortality after coronary artery bypass surgery. J Am Coll Cardiol.

[2] Peretto G, Durante A, Limite LR, Cianflone D (2014). Postoperative arrhythmias after cardiac surgery: incidence, risk factors, and therapeutic management. Cardiol Res Pract.

[3] Lahtinen J, Biancari F, Salmela E (2004). Postoperative atrial fibrillation is a major cause of stroke after on-pump coronary artery bypass surgery. Ann Thorac Surg.

[4] Maisel WH, Rawn JD, Stevenson WG (2001). Atrial fibrillation after cardiac surgery. Ann Intern Med.

[5] Narisawa A, Nakane M, Kano T (2015). Dexmedetomidine sedation during the nighttime reduced the incidence of postoperative atrial fibrillation in cardiovascular surgery patients after tracheal extubation. J Intensive Care.

[6] January CT, Wann LS, Alpert JS (2014). 2014 AHA/ACC/HRS guideline for the management of patients with atrial fibrillation: a report of the American College of Cardiology/American Heart Association Task Force on practice guidelines and the Heart Rhythm Society. Circulation.

[7] Huskisson EC (1974). Measurement of pain. Lancet.

[8] Aranki SF, Shaw DP, Adams DH (1996). Predictors of atrial fibrillation after coronary artery surgery. Current trends and impact on hospital resources. Circulation.

[9] El-Gendy HA, Dabsha MH, Elewa GM (2020). Predictors of postoperative atrial fibrillation after coronary artery bypass grafting: a prospective observational cohort study. Ain Shams J Anesthesiol.

[10] Ismail MF, El-Mahrouk AF, Hamouda TH, Radwan H, Haneef A, Jamjoom AA (2017). Factors influencing postoperative atrial fibrillation in patients undergoing on-pump coronary artery bypass grafting, single center experience. J Cardiothorac Surg.

[11] Kalman JM, Munawar M, Howes LG (1995). Atrial fibrillation after coronary artery bypass grafting is associated with sympathetic activation. Ann Thorac Surg.

[12] Ren J, Zhang H, Huang L, Liu Y, Liu F, Dong Z (2013). Protective effect of dexmedetomidine in coronary artery bypass grafting surgery. Exp Ther Med.

[13] Ueki M, Kawasaki T, Habe K, Hamada K, Kawasaki C, Sata T (2014). The effects of dexmedetomidine on inflammatory mediators after cardiopulmonary bypass. Anaesthesia.

[14] Mukhtar AM, Obayah EM, Hassona AM (2006). The use of dexmedetomidine in pediatric cardiac surgery. Anesth Analg.

[15] Tobias JD, Chrysostomou C (2013). Dexmedetomidine: antiarrhythmic effects in the pediatric cardiac patient. Pediatr Cardiol.

[16] Ling X, Zhou H, Ni Y, Wu C, Zhang C, Zhu Z (2018). Does dexmedetomidine have an antiarrhythmic effect on cardiac patients? A meta-analysis of randomized controlled trials. PLoS One.

[17] Soltani G, Jahanbakhsh S, Tashnizi MA, Fathi M, Amini S, Zirak N, Sheybani S (2017). Effects of dexmedetomidine on heart arrhythmia prevention in off-pump coronary artery bypass surgery: a randomized clinical trial. Electron Physician.

[18] Azimaraghi O, Atef Yekta R, Movafegh A (2018). The effect of dexmedetomidine on the incidence of atrial fibrillation after coronary artery bypass graft surgery. Arch Anesth Crit Care.

[19] Turan A, Bashour CA, You J, Kirkova Y, Kurz A, Sessler DI, Saager L (2014). Dexmedetomidine sedation after cardiac surgery decreases atrial arrhythmias. J Clin Anesth.

[20] Karaman Y, Abud B, Tekgul ZT, Cakmak M, Yildiz M, Gonullu M (2015). Effects of dexmedetomidine and propofol on sedation in patients after coronary artery bypass graft surgery in a fast-track recovery room setting. J Anesth.

[21] Herr DL, Sum-Ping ST, England M (2003). ICU sedation after coronary artery bypass graft surgery: dexmedetomidine-based versus propofol-based sedation regimens. J Cardiothorac Vasc Anesth.

[22] Shehabi Y, Grant P, Wolfenden H, Hammond N, Bass F, Campbell M, Chen J (2009). Prevalence of delirium with dexmedetomidine compared with morphine based therapy after cardiac surgery: a randomized controlled trial (DEXmedetomidine COmpared to Morphine-DEXCOM Study). Anesthesiology.

[23] Liu X, Zhang K, Wang W, Xie G, Fang X (2016). Dexmedetomidine sedation reduces atrial fibrillation after cardiac surgery compared to propofol: a randomized controlled trial. Crit Care.

[24] Fuller JA, Adams GG, Buxton B (1989). Atrial fibrillation after coronary artery bypass grafting. Is it a disorder of the elderly?. J Thorac Cardiovasc Surg.

[25] Asher CR, Miller DP, Grimm RA (1998). Analysis of risk factors for development of atrial fibrillation early after cardiac valvular surgery. Am J Cardiol.

[26] Zacharias A, Schwann TA, Riordan CJ, Durham SJ, Shah AS, Habib RH (2005). Obesity and risk of new-onset atrial fibrillation after cardiac surgery. Circulation.

[27] Perrier S, Meyer N, Hoang Minh T (2017). Predictors of atrial fibrillation after coronary artery bypass grafting: a Bayesian analysis. Ann Thorac Surg.

[28] Barrett TW, Abraham RL, Jenkins CA, Russ S, Storrow AB, Darbar D (2012). Risk factors for bradycardia requiring pacemaker implantation in patients with atrial fibrillation. Am J Cardiol.

[29] Heath R, Sauer WH, Aleong R, Nguyen DT (2012). Tachycardia-induced tachycardia. J Cardiovasc Electrophysiol.

[30] Rotter M, Sanders P, Takahashi Y (2004). Images in cardiovascular medicine. Coronary sinus tachycardia driving atrial fibrillation. Circulation.

